# Supplementary material for: Cellular and molecular signatures of in vivo imaging measures of GABAergic neurotransmission in the human brain
Source: Commun Biol. 2022 Apr 19;5:372. doi: 10.1038/s42003-022-03268-1 (PMC9018713; doi:10.1038/s42003-022-03268-1)
Supplement: Supplementary file 3 — Description of Additional Supplementary Files [file 42003_2022_3268_MOESM3_ESM.pdf]

Description of Additional Supplementary Files

File name: Supplementary Data 1
Description: Results from the first principal component of the gene-wise PLS regression analysis for [^{11}C]Ro15-4513.

File name: Supplementary Data 2
Description: Results from the first principal component of the cluster-wise PLS regression analysis for [^{11}C]Ro15-4513.

File name: Supplementary Data 3
Description: Results from the first principal component of the gene-wise PLS regression analysis for [^{11}C]flumazenil.

File name: Supplementary Data 4
Description: Results from the first principal component of the cluster-wise PLS regression analysis for [^{11}C]flumazenil.
