# Supplementary material for: Cellular and molecular signatures of in vivo imaging measures of GABAergic neurotransmission in the human brain
Source: Commun Biol. 2022 Apr 19;5:372. doi: 10.1038/s42003-022-03268-1 (PMC9018713; doi:10.1038/s42003-022-03268-1)
Supplement: Supplementary file 8 — Reporting Summary [file 42003_2022_3268_MOESM8_ESM.pdf]

nature research

Corresponding author(s): Paulina B. Lukow
paulina.lukow@kcl.ac.uk

Last updated by author(s): Feb 14, 2022

Reporting Summary

Nature Research wishes to improve the reproducibility of the work that we publish. This form provides structure for consistency and transparency in reporting. For further information on Nature Research policies, see our Editorial Policies and the Editorial Policy Checklist.

Statistics

For all statistical analyses, confirm that the following items are present in the figure legend, table legend, main text, or Methods section.

n/a	Confirmed	
☐	☒	The exact sample size (*n*) for each experimental group/condition, given as a discrete number and unit of measurement
☐	☒	A statement on whether measurements were taken from distinct samples or whether the same sample was measured repeatedly
☐	☒	The statistical test(s) used AND whether they are one- or two-sided<br>*Only common tests should be described solely by name; describe more complex techniques in the Methods section.*
☒	☐	A description of all covariates tested
☐	☒	A description of any assumptions or corrections, such as tests of normality and adjustment for multiple comparisons
☐	☒	A full description of the statistical parameters including central tendency (e.g. means) or other basic estimates (e.g. regression coefficient) AND variation (e.g. standard deviation) or associated estimates of uncertainty (e.g. confidence intervals)
☒	☐	For null hypothesis testing, the test statistic (e.g. F, t, r) with confidence intervals, effect sizes, degrees of freedom and P value noted<br>*Give P values as exact values whenever suitable.*
☒	☐	For Bayesian analysis, information on the choice of priors and Markov chain Monte Carlo settings
☒	☐	For hierarchical and complex designs, identification of the appropriate level for tests and full reporting of outcomes
☐	☒	Estimates of effect sizes (e.g. Cohen's *d*, Pearson's *r*), indicating how they were calculated

Our web collection on statistics for biologists contains articles on many of the points above.

Software and code

Policy information about availability of computer code

Data collection	Human gene expression microarray data was extracted from the Allen Human Brain Atlas with the abagen toolbox (https://github.com/netneurolab/abagen) in JupyterLab Notebook through anaconda3 in Python 3.8.55. The [11C]Ro15-4513 PET images were acquired on a SignaTM PET-MR General Electric (3T) scanner using the MP26 software (01 and 02). The open-access [11C]flumazenil binding parametric map was downloaded from https://xtra.nru.dk/BZR-atlas/.
Data analysis	Hierarchical clustering of genes by their expression across brain regions was performed with the WGCNA package in R 4.0.3. Pairwise correlation analysis was performed and visualised in R 4.0.3 using the Hmisc and corrplot packages. The individual parametric maps of [11C]Ro15-4513 binding were generated with MIAKAT v3413 in Matlab 2017a, averaged and reslices with SPM12's imCalc and Co-register: Reslice functions, and resampled using the fslmeants function from FSL. The same resampling method was applied to the [11C]flumazenil binding parametric map. Partial least squares regression analysis was performed using an adapted Matlab script from Whitaker et al., 2016 (https://doi.org/10.1073/pnas.1601745113) using Matlab 2017a.

For manuscripts utilizing custom algorithms or software that are central to the research but not yet described in published literature, software must be made available to editors and reviewers. We strongly encourage code deposition in a community repository (e.g. GitHub). See the Nature Research guidelines for submitting code & software for further information.

Data

Policy information about availability of data

All manuscripts must include a data availability statement. This statement should provide the following information, where applicable:

- Accession codes, unique identifiers, or web links for publicly available datasets
- A list of figures that have associated raw data
- A description of any restrictions on data availability

The datasets generated during and/or analysed during the current study are provided as Supplementary Data or available in the Figshare repository, https://doi.org/10.6084/m9.figshare.1916966376. All other source data can be accessed from the public sources used. Any other data are available from the corresponding author (or other sources, as applicable) on reasonable request.

Field-specific reporting

Please select the one below that is the best fit for your research. If you are not sure, read the appropriate sections before making your selection.

☒ Life sciences ☐ Behavioural & social sciences ☐ Ecological, evolutionary & environmental sciences

For a reference copy of the document with all sections, see nature.com/documents/nr-reporting-summary-flat.pdf

Life sciences study design

All studies must disclose on these points even when the disclosure is negative.

Sample size	The Allen Human Brain Atlas microarray dataset contains data from six healthy donors - all were included. The [11C]Ro15-4513 binding parametric map contains data on 10 healthy volunteers meeting criteria for a healthy volunteer (e.g., no history of psychiatric or neurological disorders). The open-access [11C]flumazenil binding parametric map contains data on 16 healthy volunteers - all were included. The sample sizes are comparable to those used in other studies taking a similar approach, e.g., Anderson et al., 2020 (https://doi.org/10.1038/s41467-020-16710-x) or Nørgaard et al., 2021 (https://doi.org/10.1016/j.neuroimage.2021.117878).
Data exclusions	Gene expression microarray data were subjected to intensity-based thresholding. The thresholding involved exclusion of any genes for which expression measurement did not reliably achieve level above background (probes with intensity less than background in ≥50% of samples were discarded). This resulted in exclusion of some microarray data at the point of extraction. Genes of interest which exclusion was based on this intensity-based thresholding are clearly stated in the manuscript.
Replication	The findings presented in this study were not replicated. Allen Human Brain Atlas remains the only publicly available gene expression dataset with resolution high enough to perform resampling into a brain atlas that can also be used for neuroimaging data analysis. The neuroimaging data used in the study comprised one open-access dataset and one dataset collected by the authors. Future free availability of similar datasets will enable replication of the study's findings.
Randomization	This was not relevant to this study. The study involved observational data from healthy participants only.
Blinding	This was not relevant to this study. No intervention was tested in this study.

Reporting for specific materials, systems and methods

We require information from authors about some types of materials, experimental systems and methods used in many studies. Here, indicate whether each material, system or method listed is relevant to your study. If you are not sure if a list item applies to your research, read the appropriate section before selecting a response.

Materials & experimental systems

n/a	Involved in the study	
☒	☐	Antibodies
☒	☐	Eukaryotic cell lines
☒	☐	Palaeontology and archaeology
☒	☐	Animals and other organisms
☐	☒	Human research participants
☒	☐	Clinical data
☒	☐	Dual use research of concern

Methods

n/a	Involved in the study	
☒	☐	ChIP-seq
☒	☐	Flow cytometry
☐	☒	MRI-based neuroimaging

Human research participants

Policy information about studies involving human research participants

Population characteristics	All data analysed in this study came from healthy volunteers. The Allen Human Brain Altas microarray dataset came from six donors (one female, mean age +/- SD 42.5 +/- 13.38, range 24-57). The parametric maps of [11C]Ro15-4513 binding came from 10 healthy volunteers (four females, mean age +/- SD 25.40 +/- 3.20, range 22-30). The [11C]flumazenil binding parametric map came from 16 healthy volunteers (nine females, mean age +/- SD 26.6 +/- 8, range 16-46).
Recruitment	Participants included in the [11C]Ro15-4513 scanning were recruited by public advertisement. Recruitment for the microarray and [11C]flumazenil imaging experiments was not performed by the authors of this study.
Ethics oversight	Institutional Review Board approval was granted to the Allen Institute for the microarray experiment. The [11C]Ro15-4513 scanning experiment was approved by the London/Surrey Research Ethics Committee. The [11C]flumazenil experiment was approved by the Regional Ethics Committee.

Note that full information on the approval of the study protocol must also be provided in the manuscript.

Magnetic resonance imaging

Experimental design

Design type	Structural scan
Design specifications	Not applicable - structural scan
Behavioral performance measures	Not applicable - structural scan

Acquisition

Imaging type(s)	Structural T1-weighted MRI scan
Field strength	3T
Sequence & imaging parameters	IR-FSPGR (voxel size: 1x1x1mm3, field of view=25.6, 200 slices, TR=6.992ms, TE=2.996ms, TI=400ms, flip angle=11o)
Area of acquisition	While-brain

Diffusion MRI ☐ Used ☒ Not used

Preprocessing

Preprocessing software	The structural IR-FSPGR image was preprocessed alongside the PET image with MIAKAT v3413 in Matlab 2017a. All the software preprocessing settings were MIAKAT default parameters.
Normalization	For each subject, an isotropic, skull-stripped IR-FSPGR structural image normalised to the MNI template was co-registered onto an isotropic, motion-corrected integral image created from the PET time series.
Normalization template	MNI
Noise and artifact removal	The PET acquisition was corrected for signal attenuation with a ZTE sequence (voxel size: 2.4x2.4x2.4mm3, field of view=26.4, 116 slices, TR=400ms, TE=0.016ms, flip angle=0.8o).
Volume censoring	No volume censoring was performed on the data.

Statistical modeling & inference

Model type and settings	Not applicable - structural scan taken only for co-registration purposes
Effect(s) tested	Not applicable - structural scan taken only for co-registration purposes

Specify type of analysis: ☐ Whole brain ☐ ROI-based ☐ Both

Statistic type for inference (See Eklund et al. 2016)	Not applicable - structural scan taken only for co-registration purposes
Correction	Not applicable - structural scan taken only for co-registration purposes

Models & analysis

n/a	Involved in the study
☒	☐ Functional and/or effective connectivity
☒	☐ Graph analysis
☒	☐ Multivariate modeling or predictive analysis
